# Prospective Study on Association of Prostatic Calcifications with Clinical Symptoms and Results of Treatment in Men with type III prostatitis

**DOI:** 10.1038/s41598-017-05550-3

**Published:** 2017-07-12

**Authors:** Xiang Fei, Wei Jin, Shengyu Hua, Yan Song

**Affiliations:** 1 Urology Division, Sheng Jing Hospital, China Medical University, Shenyang, 110000 China; 2 Institute of Traditional Chinese Medicine, Tianjin Key Laboratory of Chinese Medical Pharmacology, Tianjin University of Traditional Chinese Medicine, Tianjin, 300193 China

## Abstract

The purpose is to investigate the clinical significance of prostatic calculi in patients with chronic prostatitis and to discuss the possible treatment.The data from 277 young males with CP/CPPS were analyzed prospectively. Symptom severity was measured using the National Institutes of Health Chronic Prostatitis Symptom Index (NIH-CPSI) and the International Prostatic Symptoms Score (IPSS). Sexual function was assessed by the International Index of Erectile Function (IIEF-5) questionnaire. After four weeks of therapy, the NIH-CPSI, IPSS, and IIEF-5 tests were repeated. The variables were compared between patients with and without prostatic calcifications using the Students t-test or chi-square test. No significant differences were found between CP/CPPS patients with and without prostatic calcifications regarding age, body mass index, prostate volume, CPSI, IPSS and IIEF-5. Men with calcifications endured symptoms significantly longer (37.9 ± 25.2 versus 19.0 ± 16.4 months, P < 0.01), and had significantly higher white blood cell counts per high power field in expressed prostatic secretions (7.7 ± 12.8 versus 3.9 ± 4.7; P < 0.01), than patients without prostatic calcifications, who responded better to medication compared with patients with prostatic calcifications. In conclusion, patients with calcifications were more likely to have category IIIA disease and they required a longer medication period.

## Introduction

Category III prostatitis, or chronic prostatitis/chronic pelvic pain syndrome, (CP/CPPS) is a common yet poorly understood condition. The prevalence of CP/CPPS has been estimated to be over 90% of all chronic prostatitis patients^1^.

Prostatitis produces lower urinary tract symptoms (LUTS) by causing contraction of the smooth muscle of the prostate and bladder neck, and also causes chronic pelvic pain^2^. Moreover, CP/CPPS is associated with significant sexual dysfunction, including erectile dysfunction (ED), decreased sexual desire, and decreased frequency of sexual activities^3–5^.

The prostatic calcifications are identified by transrectal ultrasound (TRUS)during clinical practice from time to time. Some authors suggest that prostatic calculi simply accompany the presence of prostatic hyperplasia and carcinoma^6, 7^. However, some studies have correlated the presence of prostatic calcifications with CP/CPPS-related symptoms^8, 9^. Histology reveals that most calculi are associated with inflammatory changes. Intraprostatic urinary reflux, which causes chemical prostatitis, has an important role in the pathogenesis of nonbacterial prostatitis and prostatodynia^10^. It is also reported that prostatic calculi are formed by the precipitation of prostatic secretions and calcification of the corpora amylacea under inflammatory conditions^11^.

There have been several studies concerning the influence of prostatic calculi on LUTS or CP/CPPS–related symptoms, with conflicting results^2, 8, 12^; therefore, it remains unclear whether different treatment should be applied to chronic prostatitis patients with calculi versus those without calculi.

The aim of this study was to investigate the incidence and clinical significance of prostatic calculi in young adults with CP/CPPS and to explore possible treatments for CP/CPPS with prostatic calculi.

## Results

All 277 CP/CPPS patients were evaluated. 5 patients were excluded from the study due to lost follow-ups.A total of 121 patients had significant calcifications within the prostate. Thus, the incidence of prostatic calcification was 43.7% in our series. As seen in Table 1, no significant differences were found between CP/CPPS patients with and without prostatic calcifications regarding age, BMI, and prostate volume. The calcification group had CPSI, IPSS, and IIEF-5 scores similar to those of the no-calcification group. Men with calcifications endured symptoms for significantly longer time (37.9 ± 25.2 versus 19.0 ± 16.4 months, P < 0.01).

**Table 1:** Clinical variables between patients with and without prostatic calculi.

variables	CP with PC (n = 121)	CP without PC (n = 151)	P value
age	35.4 ± 7.3	33.8 ± 7.4	0.873
BMI	24.9 ± 3.8	25.2 ± 4.0	0.155
Duration	37.9 ± 25.2	19.0 ± 16.4	0.001
Prostate volume	27.4 ± 4.4	27.1 ± 4.9	0.515
IPSS	15.6 ± 6.6	15.3 ± 7.0	0.496
NIH-CPSI	23.3 ± 7.1	23.1 ± 6.9	0.767
IIEF-5	15.8 ± 4.7	15.7 ± 4.8	0.729
WBC in EPS (counts/hpf)	7.7 ± 12.8	3.9 ± 4.7	0.001

*Compared the value between the calcification and no calcification groups

BMI = body mass index; CP = chronic prostatitis; PC = prostate calification; IPSS = international prostate symptom score; hpf = high power field; NIHCPSI = National Institutes of Health Chronic Prostatitis Symptom Index; IIEF = International Index of Erectile Function; EPS = expressed prostatic secretion; WBC = white blood cell

Data are presented as means ± standard deviation,

All patients received a same combination treatment.Treatment received at bedtime consisted of: 1) α-blocker(tamsolusin, 2 mg po); 2) an herbal supplement of pollen extract; 3) a rectal suppository containing a proprietary blend of grape seed extract and hawthorn berry; and 4) antibiotics(Levofloxacin, 500 mg po). that were administered for 4 weeks, if Type IIIA prostatitis occurred.

At the four-week interval of therapy, the NIH-CPSI, IPSS, and IIEF-5 tests were repeated and the results were compared between the calcification group and no calcification group.

As shown in Table 1, men with prostatic calcifications had significantly higher white blood cell counts per high power field in EPS (7.7 ± 12.8 versus 3.9 ± 4.7; P < 0.01). Defining EPS inflammation as at least 10 white blood cells (WBCs) per high power field (hpf), 69 (24.9%) men had category IIIA (inflammatory) disease and 203 (75.1%) men had category IIIB disease (non-inflammatory). Men in the calcification group had significantly higher WBC counts/hpf in EPS and were more likely to have category IIIA disease (P < 0.001) (Table 2).

**Table 2:** comparison of subtpe of Type III CPPS patients with and without prostatic calcification(P < 0.01).

variables	CP with PC (n = 121)	CP without PC (n = 151)	Total
III a	45	24	69
III b	76	127	203
Total	121	151	

CP = chronic prostatitis; PC = prostate calification

The analysis of variance and paired t-test were used to analyze characteristics. Categorical variables were compared using the Pearson chi-square test. P < 0.05 was considered statistically significant.

CP/CPPS patients without prostatic calcifications responded better to medication compared with patients with prostatic calcifications, as shown in Table 3. In the no-calcifications group, the NIH-CPSS and IPSS dropped significantly after 8 weeks of medication compared with those before medication; however, sexual function improved after 12 weeks of medication. In the calcifications group, it took at least 12 weeks to observe symptom relief, whereas sexual function showed no improvement, even after 12 weeks. These data indicated that a longer time of medication was required for symptom improvement in the patients with prostatic calcifications.

**Table-3:** Comparison of IPSS, NIH-CPSI and IIEF-5 scores in patients with and without prostatic calcification after treatment,

variables	CP with PC (n = 121)				CP without PC (n = 151)			
	0 week	4 weeks	8 weeks	12 weeks	0 week	4 weeks	8 weeks	12weeks
IPSS	15.6 ± 6.6	15.4 ± 6.1	15.8 ± 5.8	12.5 ± 4.7*	15.3 ± 7.0	14.3 ± 6.2	12.8 ± 5.2*	11.8 ± 4.8
NIH-CPSI	23.3 ± 7.1	23.0 ± 6.6	21.5 ± 6.0*	18.5 ± 5.3	23.1 ± 6.9	22.9 ± 6.5	19.1 ± 6.2*	17.0 ± 5.8
IIEF-5	15.8 ± 4.7	15.9 ± 4.3	16.2 ± 4.1	16.7 ± 4.1	15.7 ± 4.8	15.8 ± 4.7	16.4 ± 4.4	18.3 ± 4.3*
WBC	7.7 ± 12.8	7.6 ± 11.3	6.7 ± 8.8	6.9 ± 8.6	3.9 ± 4.7	3.9 ± 4.7	3.5 ± 3.7	2.9 ± 2.7

CP = chronic prostatitis; PC = prostate calification; IPSS = international prostate symptom score; NIH-CPSI = National Institutes of Health Chronic Prostatitis Symptom Index; IIEF = International Index of Erectile Function

WBC = white blood cell.

The analysis of variance and paired t-test were used to analyze characteristics. Categorical variables were compared using the Pearson chi-square test. P < 0.05 was considered statistically significant. The result were compared with the a base line 0 week time point groups before treatment.Data are presented as means ± standard deviation(*P < 0.05)

## Discussion

Prostatic calculi are encountered frequently in urological practice; it is unknown whether prostatic calculi are clinically insignificant or whether they have the potential to cause symptoms. In this retrospective, descriptive study, we wished to characterize the incidence and the clinical features of prostatic calculi in men with type III prostatitis.

There may be different incidences of prostatic calculi owing to divergences in definition and in the populations studied^13^. Geramoutsos *et al* ^8^. screened 1374 men younger than 50 years old and found 101 (7.4%) cases of prostatic stones. Park and his colleagues2 identified the presence of prostatic calculi in 41.8% of 802 men who complained of LUTS. The incidence of prostatic calcifications in our series was different from these reports; this disparity is likely due in part to different patient populations, and to differences in detection rates of prostatic calculi, which depends on the sensitivity of the imaging method. Abdominal ultrasound was used to detect the prostate calcification in Geramoutsos’s study^8^. In addition, definition and classification of prostatic calcifications were not standardized.

There are two types of calcification according to the echo patterns of prostatic calculi, as previously described: type I: discrete, multiple small echoes, usually diffusely distributed throughout the gland and type II: large mass of multiple, coarser echoes^14^.

**Figure 1:**
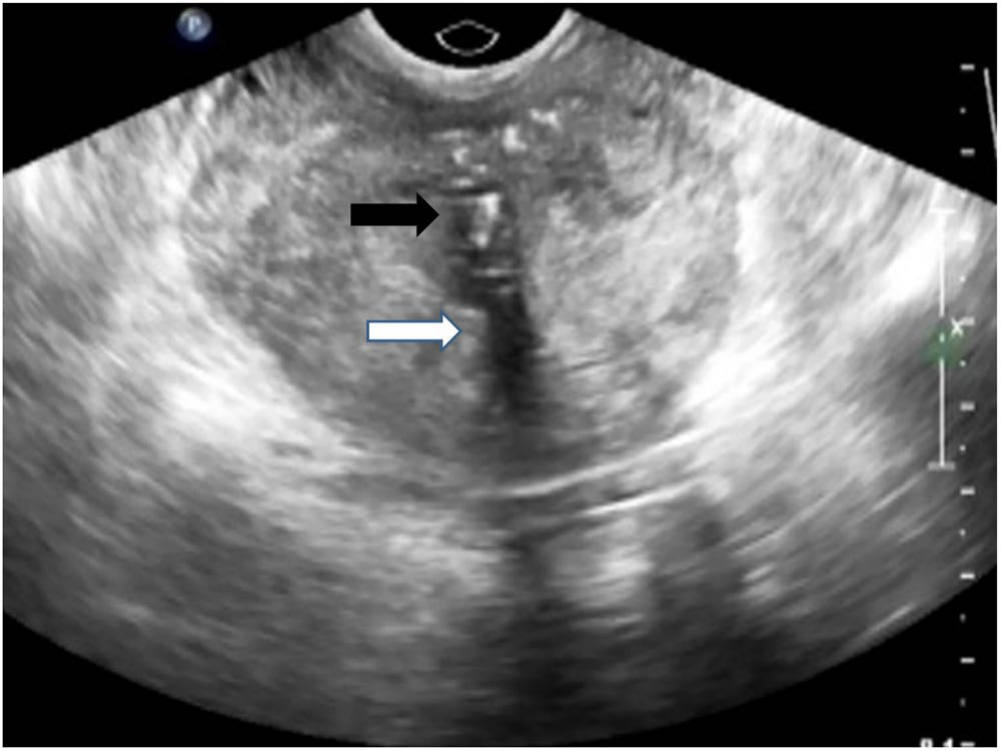
Prostatic calcifications with acoustic shadowing. Legend: The ultrasound image of patients with prostate calcification on the first clinic visit The prostate calcification appeared as the hyper echogeneous foci (black arrow). The acoustic shadowing appeared as the dark tail (white arrow)

In this study, only the larger (with the diameter over 3 mm), more echogenic foci (Fig. 1) that caused acoustic shadowing were considered significant prostatic calcification. Sung-Woo Park^15^ and Daniel^16^ reported that prostatic inflammatory changes were closely associated with type II calcification. These calculi are usually larger, situated mainly in the prostatic ducts and their composition is similar to stones found anywhere in the urinary tract^17, 18^. The discrete calculi with multiple small echoes, which diffusely distributed throughout the gland were considered as a normal change of aging with no clinical significanceand were not included in the study^19^.

The exact mechanisms of calcification formation in the prostate remain unknown; calcifications are usually distributed throughout the entire prostate gland but are more frequently observed in the transition zone than in other zones^20^ Current data suggests calculi are usually multifaceted and situated mainly in the prostatic ducts^21^.In this study, most of the prostate calcification(90.6%) were observed in the transition zone. And there was no significant difference in the effectiveness of therapy based on the location of the prostate (data not shown in the paper)

Prostatic calculi are common in patients with CP/CPPS and are associated with greater inflammation and symptoms^22^. Previous study has shown that most calculi are associated with histological inflammatory changes: inflammation infiltration of lymphocytes and histiocytes is closely related to prostatic lithiasis^23^. Dilatation of the prostatic duct and urinary reflux may be the possible mechanism in the development of calculi. The composition of prostate calcification cannot be found in prostatic secretions but it is similar to stones in the urinary tract^17, 24^. Arnaud and his colleagues suggested that long-term infection has a significant role in the lithogenic process of prostate calcification and bacterial imprints were discovered on prostate calcification^25^.

They discovered a high occurrence of bacterial imprints (78%) in 23 prostatic stones, which indicated a past or present infection of the prostate tissue. Another study showed that therapy designed to medically dissolve the stones led to symptomatic improvement^11^ Our patients with calcification had increased markers of inflammation in their EPS, a finding seen by others^8, 26^. Ludwig *et al*. concluded that prostatic calculi are typical signs of inflammation^27^. However, Sung-Woo Park *et al* ^15^. did not show that prostatitis caused prostatic calculi. Their study showed a significant difference in the duration of pelvic pain between prostatic calculi and noncalculi groups, also confirmed by our study, but did not show a significant difference in the WBC count of prostatic fluids.

Although this study did not reveal the association of ED of patients with and without calcifications; patients without prostate calcification responded better to medication. There may be several underlying mechanisms to explain this. First, prostatic inflammation affects smooth muscle relaxation and impairs microvascularization of the prostate^28^, thus decreasing the ability of penile tissue to maintain an erection. Second, inflammation of the prostate might impair chemokine, nitric oxide synthase, and cyclooxygenase-2 production^29^. Furthermore, inflammation- related pelvic floor spasm may cause the onset of erectile dysfunction^30^.

To our knowledge, our study is the first to compare the effects of medication in CP/ CPPS patients with and without prostate calculi. Our data suggests that the presence of calcifications correlates with a longer effective treatment period, suggesting a possible chronic infection. A possible mechanism whereby CP/CPPS becomes chronic and resistant to medication is calcification; chronic calcification could lead to local tissue injury and inflammation, and the calcification itself could be the source of infection that harbors microorganisms. A recent study demonstrated that men with recalcitrant CPPS with prostatic calculi treated by combination therapy to eradicate nanobacterial calcification improved significantly after three months^11^.

In this study, prostatic calcification was associated with longer symptom duration. If calcification is an effect of repeated bouts of infection and/or inflammation, calcification could be a marker of disease duration. The obstruction of prostatic glands duct could be caused by calcification, which can lead to increased intraprostatic pressures and secondary inflammation. Such a mechanism could explain the temporary relief of symptoms by anti-inflammatory medication and prostatic massage. Persistent inflammation in the area could lead to persistent nerve and muscle irritation, resulting in pain and lower urinary tract symptoms. Moreover, in this situation, calcification might indicate the later chronic stage of the disease which monotherapyis not effective^31^.

The limitations of this study included a lack of ultrasonography data regarding the size and location(s) of calcifications; however, the criteria for defining and classifying prostatic calcifications have yet to be well established. We anticipate that larger studies in the future will better characterize subtypes of prostatic calculi and will help to evaluate the association between prostatic lithiasis and CP/CPPS. If calcification is to be a marker of chronicity then surely it must be possible to quantify the degree of calcification by Transrectal Ultrasound. It would be useful to have a grading which would allow immediate identification of the patient least likely to respond. In this study, larger foci(over 3 mm) that caused acoustic shadowing were considered prostatic calcifications. Despite these limitations, this study presents the first comparison of the clinical significance of CP/CPPS in patients with and without prostatic calcifications and adds important knowledge to inform the design of treatment studies.

Our results indicated that patients with calcifications endured symptoms for significantly longer time and were more likely to have the type IIIA prostatitis compared to patients without calcifications. A longer medication period was required in the patients with prostatic calcifications for optimal treatment results.

## Materials and Methods

### Patient selection

The study was conducted between December 2012 and January 2014, after approval from the Institutional Review Board (Ethic Committee of Medical Research and New Technology of ShengJing hospital); 277 males patient with a diagnosis of CP/CPPS were prospectively evaluated in the urology and andrology clinic of our hospital. Authors had no access to information that could identify individual participants during or after data collection. All clinical investigations were conducted according to the principles expressed in the Declaration of Helsinki. Informed consent was obtained from all participants and/or their legal guardians.

The patients complained primarily of urological voiding and pain, as well as sexual dysfunction. All patients underwent a complete history, physical examination, and culture of urine and expressed prostatic secretions (EPS). All patients were diagnosed with type III prostatitis (CP/CPPS) according to the National Institutes of Health (NIH) criteria^32^.

Exclusion criteria were based on the NIDKK approved criteria for studies on chronic prostatitis. Patients over 50 years old were also excluded from the study to avoid measuring symptoms and calcification due to benign prostatic hyperplasia. Patients who lost follow-up were also excluded from the study.

TRUS was performed using an 8.0-MHz rectal probe (GE Healthcare, LOGIQ P6-PRO, Little Chalfont, UK). The prostate volume (PV) was measured by TRUS using the formula for an elliptic volume. Only the larger, more echogenic foci (Fig. 1) that caused acoustic shadowing were considered prostatic calcifications in this study. According to the presence of prostatic calcifications, we prospectively divided the patients into a calcification group and a no-calcification group for the analysis of the result of treatment.

The presence of >10 leukocytes in the EPS and a post-prostatic massage urine specimen classified as voided bladder urine-3 (VB3) was categorized as the inflammatory subtype of CP/CPPS (Type IIIA). At each visit, patients had their symptoms measured by the NIH Chronic Prostatitis Symptom Index (CPSI) and International Prostatic Symptoms Score (IPSS). Sexual function was assessed by using the updated five-item International Index of Erectile Function (IIEF-5) questionnaire^33^.

Statistical analysis was performed using the statistical software, SPSS Version 14.0 for Windows (SPSS, Inc., Chicago, IL). The analysis of variance and paired t-test were used to analyze characteristics. Categorical variables were compared using the Pearson chi-square test. P < 0.05 was considered statistically significant.

### Data Availability

The datasets generated during and/or analysed during the current study are available from the corresponding author on reasonable request.

## References

[1] Schaeffer AJ (2002). Leukocyte and bacterial counts do not correlate with severity of symptoms in men with chronic prostatitis: the National Institutes of Health Chronic Prostatitis Cohort Study. J Urol.

[2] Schaeffer AJ (2002). Demographic and clinical characteristics of men with chronic prostatitis: the National Institutes of Health Chronic Prostatitis Cohort Study. J Urol.

[3] Chung SD, Keller JJ, Lin HC (2012). A case-control study on the association between chronic prostatitis/chronic pelvic pain syndrome and erectile dysfunction. BJU Int.

[4] Sonmez NC (2011). Sexual dysfunction in type III chronic prostatitis (CP) and chronic pelvic pain syndrome (CPPS) observed in Turkish patients. Int Urol Nephrol.

[5] Liang CZ (2010). Prevalence of premature ejaculation and its correlation with chronic prostatitis in Chinese men. Urology.

[6] Lee SE (2003). Prostatic calculi do not influence the level of serum prostate specific antigen in men without clinically detectable prostate cancer or prostatitis. J Urol.

[7] Sondergaard G, Vetner M, Christensen PO (1987). Prostatic calculi. Acta Path Microbiol Immunol Scand A.

[8] Geramoutsos I (2004). Clinical correlation of prostatic lithiasis with chronic pelvic pain syndromes in young adults. Eur Urol.

[9] Hong CG (2012). The prevalence and characteristic differences in prostatic calcification between health promotion center and urology department outpatients. Korean J Urol.

[10] Kirby RS, Lowe D, Bultitude MI, Shuttleworth KE (1982). Intraprostatic urinary reflux: An aetiological factor in abacterial prostatitis. Br J Urol.

[11] Shoskes DA, Thomas KD, Gomez E (2005). Anti-nanobacterial therapy for men with chronic prostatitis/chronic pelvic pain syndrome and prostatic stones: preliminary experience. J Urol.

[12] Shoskes DA (2007). Incidence and significance of prostatic stones in men with chronic prostatitis/chronic pelvic pain syndrome. Urology.

[13] Kovi J, Rao MS, Heshmat MY, Akberzie ME, Jackson MA (1979). Incidence of prostatic calcification in blacks in Washington, D.C., and selected African cities. correlation of specimen roentgenographs and pathologic findings. cooperative prostatic research group. Urology.

[14] Harada K, Igari D, Tanahashi Y (1979). Gray scale transrectal ultrasonography of the prostate. J Clin Ultrasound.

[15] Park SW, Nam JK, Lee SD, Chung MK (2010). Are prostatic calculi independent predictive factors of lower urinary tract symptoms?. Asian Journal of Andrology.

[16] Shoskes DA, Lee CT, Murphy D, Kefer J, Hadley MW (2007). Incidence and Significance of Prostatic Stones in Men with Chronic Prostatitis/ Chronic Pelvic Pain Syndrome.Urology.

[17] Meares EM (1974). Infection stones of the prostate gland. Urology.

[18] Fox M (1963). The natural history and significance of stone formation in the prostate gland. J Urol.

[19] Leader AJ, Queen DM (1958). Prostatic calculous disease. J Urol.

[20] Jae HS (2008). Calcifications in prostate and ejaculatory system: a study on 298 consecutive whole mount sections of prostate from radical prostatectomy or cystoprostatectomy specimens. Annals of Diagnostic Pathology.

[21] Peeling WB, Griffiths GJ (1984). Imaging of the prostate by ultrasound. J Urol.

[22] Zhao ZG, Jun X (2014). Prospective study on association of prostatic calcifications with sexual dysfunction in men with chronic prostatitis/chronic pelvic pain syndrome (CP/CPPS). J Sex Med.

[23] Moore RA (1936). Morphology of prostatic corpora amylacea and calculi. Arch Pathol.

[24] Sutor DJ, Wooley SE (1974). The crystalline composition of prostatic calculi. Br J Urol.

[25] Arnaud D, Paul M, Dominique B, Michel D (2012). Prostatic stones: evidence of a specific chemistry related to infection and presence of bacterial imprints. PLoS ONE.

[26] Bock E (1989). Calcifications of the prostate: a transrectal echographic study. Radiol Med.

[27] Ludwig M, Weidner W, Schroeder-Printzen I, Zimmermann O, Ringert RH (1994). Transrectal prostatic sonography as a useful diagnostic means for patients with chronic prostatitis or prostatodynia. Br J Urol.

[28] Cho IR, Keener TS, Nghiem HV, Winter T, Krieger JN (2000). Prostate blood flow characteristics in the chronic prostatitis/pelvic pain syndrome. J. Urol.

[29] Eryildirim B (2010). The effectiveness of sildenafil citrate in patients with erectile dysfunction and lower urinary system symptoms and the significance of asymptomatic inflammatory prostatitis. Int J Impot Res.

[30] Anderson RU, Wise D, Sawyer T, Chan CA (2006). Sexual dysfunction in men with chronic prostatitis/chronic pelvic pain syndrome: improvement after trigger point release and paradoxical relaxation training. J Urol.

[31] Nickel JC (2004). Failure of a monotherapy strategy for difficult chronic prostatitis/chronic pelvic pain syndrome. J Urol.

[32] Litwin MS (1999). A.The national institutes of health chronic prostatitis symptom index: development and validation of a new outcome measure. J Urol.

[33] Rosen RC, Cappelleri JC, Gendrano N (2002). The international index of erectile function (IIEF): a state-of-the-science review. Int J Impot Res.

